# Editorial: Health monitoring should reflect population diversity

**DOI:** 10.25646/6072

**Published:** 2019-09-18

**Authors:** Bärbel-Maria Kurth, Oliver Razum

**Affiliations:** 1 Robert Koch Institute, Berlin; 2 Bielefeld University

Germany is a migration country. In 2018, 23.6% of the population had a migration background [1], which means that they, or at least one of their parents, were born without German citizenship. People with a migration background are heterogeneous in regard to their opportunities, their socioeconomic situation, their language skills, the reasons they migrated to Germany and their healthcare needs. Any analysis of migration and health must be sure to take these factors into account.

The Robert Koch Institute (RKI) is currently expanding its health monitoring activities, which have been developed over the last few years, and its health reporting system, which has been established for many years. People with a migration background should be included in national health surveys according to their proportion of the population in order to enable representative statements on their health status. Here, accounting for the diversity of people with a migration background and simultaneously ensuring comparability of different data sources presents a significant challenge.

The inclusion of people with a migration background in the health surveys conducted by the RKI has been successful in the context of both the baseline study of the German Health Interview and Examination Survey for Children and Adolescents (KiGGS) and its follow-up KiGGS Wave 2 due to the application of specific measures [2, 3]. To date, this has been implemented only partial in regards to the adult population. The German Health Update (GEDA), for example, which is conducted at regular intervals, has not yet been able to provide reliable data on people with a migration background, a problem largely due to the small number of cases. In the German Health Interview and Examination Survey for Adults (DEGS1), people with a migration background were not sufficiently included [4]. As a result, the Improving Health Monitoring in Migrant Populations (IMIRA) project was initiated in 2016 with the aim of both expanding health monitoring at the Robert Koch Institute to include people with a migration background and further developing health reporting on migration and health. The IMIRA project will be concluded at the end of 2019 [5].

In this issue of the Journal of Health Monitoring, we provide results from the IMIRA project with a focus on the expansion of health reporting. The first article presents The health of children and adolescents with a migration background in Germany. The second article, Health reporting on people with a migration background – Selection and definition of (core) indicators, describes the process of developing a set of (core) indicators and its results. This article is the conceptional basis for the selected indicators in the focus article.

The third article in this issue, Concepts for migration-sensitive health monitoring, focuses on the further development and review of relevant migration-related concepts (e.g. acculturation and discrimination). The primary aim here is to better assess and depict the diversity of the German resident population in the long-term, thereby also advancing health research in the context of migration.

As well as further developing health reporting and the content-related concepts used in surveys, the IMIRA project has developed strategies to works towards the long-term inclusion of people with a migration background into health monitoring. For this, two feasibility studies were conducted to test innovative approaches to recruiting participants for interview surveys and overcoming language barriers in examination surveys [5, 6]. The results confirm [7, 8] that multilingual materials and face-to-face interviews are very helpful when trying to improve the inclusion of hard-to-reach populations. The response rates among people with limited German-language skills in examination surveys can be increased with the utilisation of a video interpreting service and multilingual information videos.

The insights presented in the articles of this issue, and the findings from the feasibility studies, allow us to identify the following factors as fundamental to a diversity-oriented health monitoring: Firstly, the use of multilingual and diversity-sensitive materials is important as is the provision of different data collection formats, e.g. online, in writing, or face-to-face interviews. Personal contact is very effective in increasing the response rates of people with a migration background in order to motivating them to participate in a study or also to conduct an interview. Language mediation via a video interpreting service is a practical way to overcome language barriers during examination surveys. Increasing diversity-sensitivity in staff training sessions, in the development of materials and in the identification of relevant concepts is essential to establishing responsible and discrimination-free health reporting on migration and health. Actively engaging people with a migration background either as staff or as external advisers within the research and reporting process helps to increase both the understanding of potential barriers and reservations and the ability to face them.

The upcoming nationwide interview and examination survey for adults, the Health and Nutrition Survey in Germany (gern survey), which will be conducted by the Robert Koch Institute in cooperation with the Max Rubner Institute in 2020, aims to apply the insights gained from the IMIRA project on how to better reach people with a migration background. It is also essential for the future development of health monitoring that these factors will be taken into account and be continuously developed, in order to do justice to the diversity of the German population and adequately reflect the health of all people living in Germany. We hope that the findings presented in this issue will be of use to other studies in the future, and we appreciate your interest.
